# Supplementary material for: Ontogenetic origins of cranial convergence between the extinct marsupial thylacine and placental gray wolf
Source: Commun Biol. 2021 Jan 8;4:51. doi: 10.1038/s42003-020-01569-x (PMC7794302; doi:10.1038/s42003-020-01569-x)
Supplement: Supplementary file 2 — Supplementary Information [file 42003_2020_1569_MOESM2_ESM.pdf]

Ontogenetic origins of cranial convergence between the extinct marsupial thylacine and placental grey wolf

Supplementary Information

Communications Biology

Axel H Newton[1,2,4 *], Vera Weisbecker[3], Andrew J Pask[2,4 #] & Christy A Hipsley[2,4 # *]

1. School of Biomedical Sciences, Monash University, Victoria, Australia
2. School of BioSciences, The University of Melbourne, Victoria, Australia
3. College of Science and Engineering, Flinders University, South Australia, Australia
4. Department of Sciences, Museums Victoria, Victoria, Australia

Supplementary figures and tables

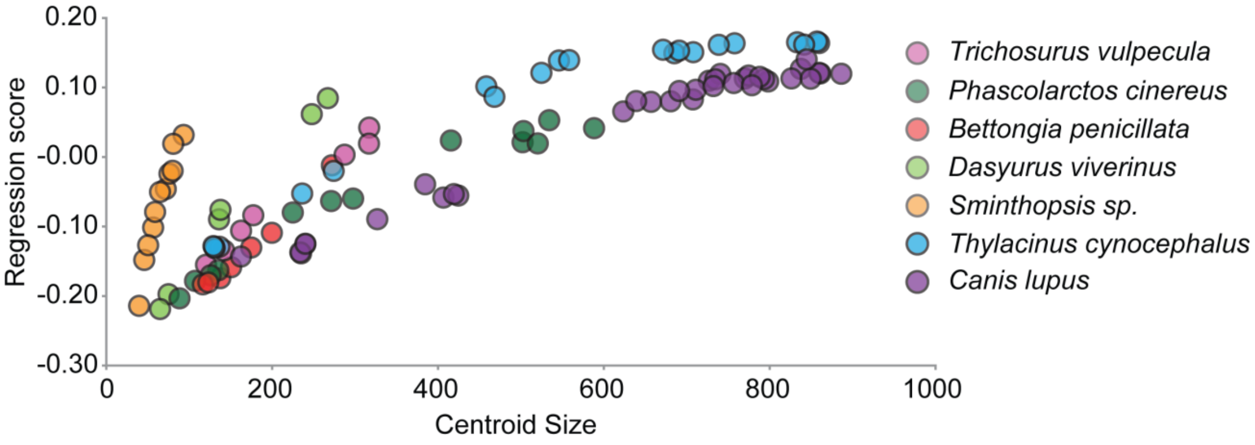

Figure S1. Ontogenetic regression of cranial shape vs centroid size. Each sampled taxa revealed variation between developmental trajectories, though the thylacine and wolf display similar gradual patterns of shape change.

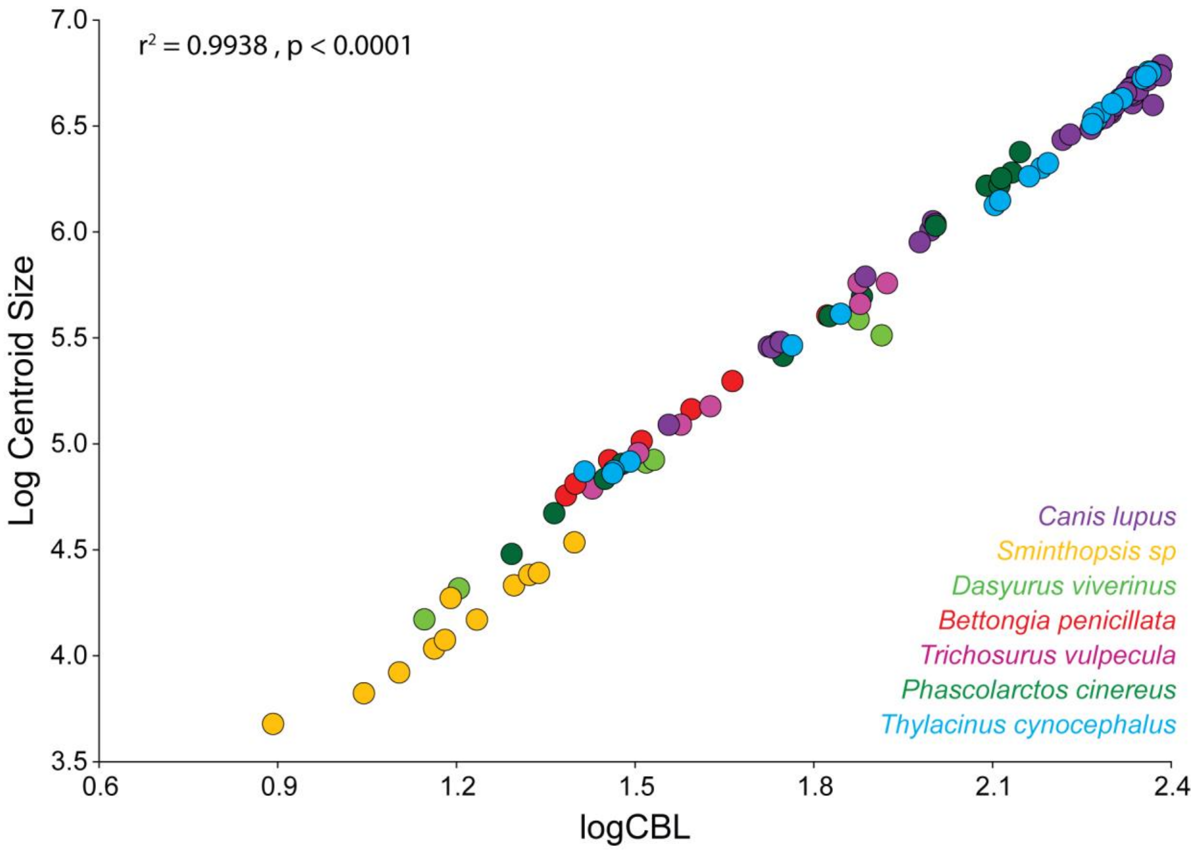

Figure S2. Regression of log transformed CBL length (in mm) vs log transformed centroid size. Skull length and centroid size are highly correlated and significant.

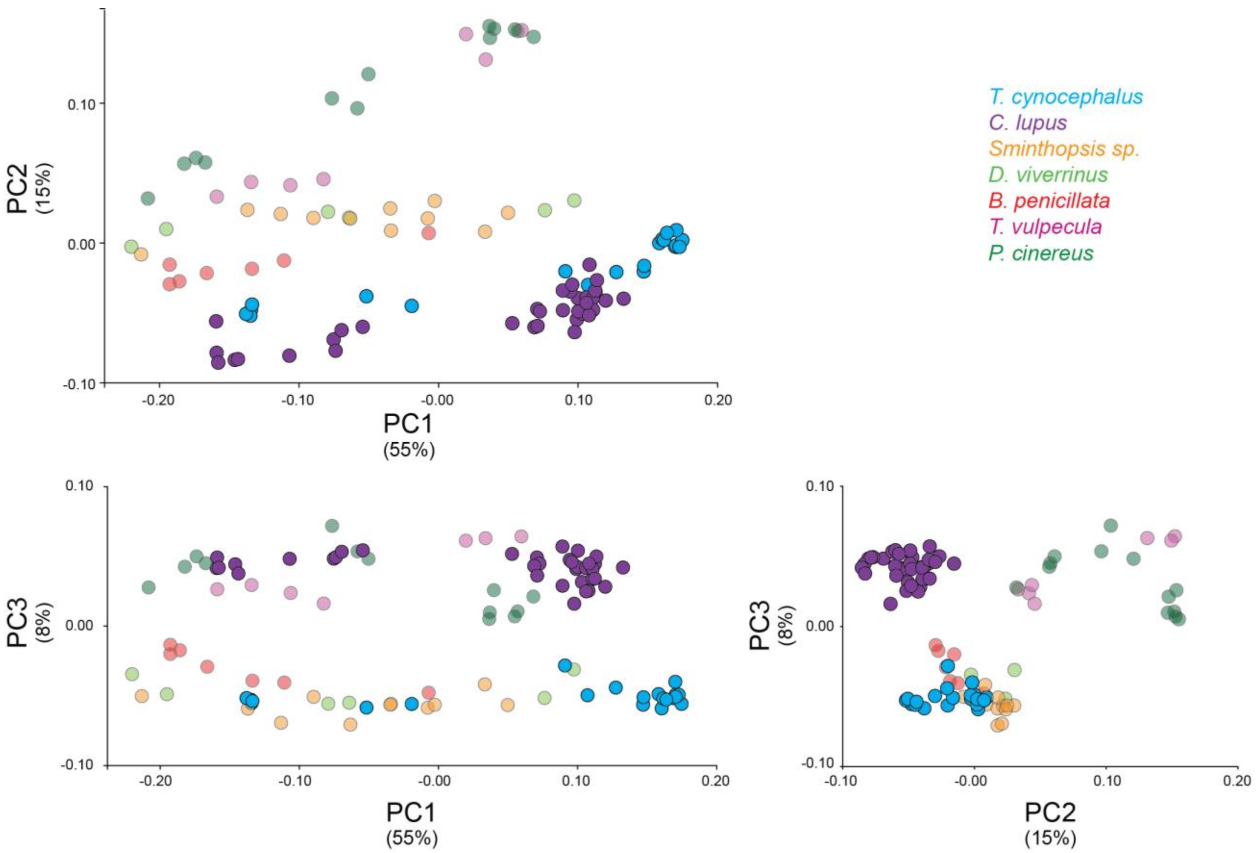

Figure S3. Extended ontogenetic morphospace showing cranial shape disparity in PC1 vs PC2 (as seen in Figure 1) as well as PC1 vs PC3 and PC2 vs PC3.

Species	n	SS	MS	R^2	F	Z	P
Bettongia penicillata	5	0.04	0.04	0.78	17.89	3.19	**0.001**
Canis lupus	27	0.39	0.39	0.78	113.90	5.10	**0.001**
Dasyurus viverinus	6	0.10	0.10	0.87	25.98	2.74	**0.015**
Phascolarctos cinereus	5	0.20	0.20	0.79	41.66	4.04	**0.001**
Sminthopsis sp.	10	0.08	0.08	0.71	21.61	3.94	**0.001**
Thylacinus cynocephalus	18	0.34	0.34	0.89	151.25	4.56	**0.001**
Trichosurus vulpecula	6	0.08	0.08	0.79	18.52	2.90	**0.002**

Table S1. Statistics from species-specific allometric regressions.

Module pair	Neonate		Juvenile		Adult	
	Thylacine	Wolf	Thylacine	Wolf	Thylacine	Wolf
	n=6	n=6	n=6	n=8	n=9	n=19
FNP - MES	0.99	0.95	0.99	1.01	0.85	0.77
FNP - PA	1.00	0.93	0.98	1.01	0.89	0.71
MES - PA	1.00	0.98	0.96	1.00	0.92	0.85
Oral/nasal - Molar	1.00	0.94	0.99	1.01	0.94	0.76
Oral/nasal - Zygo/Ptery	1.00	0.86	0.97	1.00	0.95	0.68
Oral/nasal - Orbitofrontal	1.00	0.93	0.99	1.03	0.91	0.62
Oral/nasal - Cranial vault	1.01	0.95	0.94	0.96	0.67	0.67
Oral/nasal - Basicranium	1.05	0.94	1.01	1.01	0.74	0.61
Molar - Zygo/Ptery	0.98	0.92	0.94	0.99	0.93	0.82
Molar - Orbitofrontal	1.01	0.98	0.99	1.01	0.89	0.84
Molar - Cranial vault	0.98	0.96	0.96	0.96	0.54	0.59
Molar - Basicranium	1.07	0.91	0.96	1.00	0.79	0.74
Zygo/Ptery - Orbitofrontal	1.00	0.94	0.92	1.01	0.83	0.67
Zygo/Ptery - Cranial vault	1.01	0.89	0.91	1.01	0.73	0.54
Zygo/Ptery - Basicranium	1.03	0.89	1.03	0.99	0.75	0.64
Orbitofrontal - Cranial vault	1.01	0.97	0.96	0.99	0.59	0.72
Orbitofrontal - Basicranium	1.06	0.94	0.98	1.00	0.79	0.78
Cranial vault - Basicranium	1.03	0.99	0.93	0.93	0.38	0.58

Table S2. Pairwise covariance ratio (CR) coefficients between cranial landmark modules for thylacine and wolf at neonate, juvenile, and adult developmental stages (see Fig. 1a, Table S1 for sampling). CR values < 0.85 indicated modularity, following [1,2] and values > 0.85 are suggested to be integrated. All comparisons were significant at $P \leq 0.001$. Values are visualized on thylacine and wolf skulls in Figure 4.

Module pair	Neonate		Juvenile		Adult	
	**Thylacine**	**Wolf**	**Thylacine**	**Wolf**	**Thylacine**	**Wolf**
	n=6	n=6	n=6	n=8	n=9	n=19
Oral/nasal - Molar	1.02	0.86	0.99	0.97	0.94	0.80
Oral/nasal - Zygo/Ptery	1.00	0.95	0.94	0.89	0.97	0.68
Oral/nasal - Orbitofrontal	1.00	0.98	0.93	0.94	0.98	0.69
Oral/nasal - Cranial vault	1.01	0.96	0.96	0.81	0.64	0.69
Oral/nasal - Basicranium	0.85	0.89	0.95	0.89	0.87	0.65
Molar - Zygo/Ptery	1.00	0.87	0.94	0.86	0.87	0.82
Molar - Orbitofrontal	1.00	0.95	0.95	0.93	0.88	0.84
Molar - Cranial vault	1.00	0.94	0.92	0.79	0.59	0.59
Molar - Basicranium	0.88	0.98	0.96	0.89	0.83	0.79
Zygo/Ptery - Orbitofrontal	1.00	0.99	0.93	0.89	0.91	0.62
Zygo/Ptery - Cranial vault	1.01	0.94	0.84	0.98	0.64	0.54
Zygo/Ptery - Basicranium	0.92	0.94	1.03	0.88	0.84	0.68
Orbitofrontal - Cranial vault	0.99	0.97	0.77	0.86	0.52	0.72
Orbitofrontal - Basicranium	0.81	0.96	0.99	0.88	0.89	0.75
Cranial vault - Basicranium	0.91	1.00	0.78	0.68	0.43	0.59

Table S3. Allometry-free pairwise co-variance ratio (CR) coefficients between cranial landmark modules for thylacine and wolf at neonate, juvenile, and adult developmental stages.

Supplementary data 1. List of specimens, accession IDs, skull lengths (CBL, mm), dentition patterns and corresponding stages. Specimens were obtained from published [3–8], publically available and in-house sources.

Supplementary data 2. Cranial landmark locations and module hypotheses from [9].

Supplementary data 3. Raw ontogenetic landmark coordinates.

Supplementary data 4. Associated classifier variables for raw landmark coordinates.

Supplementary data 5. Associated covariates for raw landmark coordinates.

Supplementary references

1. Bardua, C., Wilkinson, M., Gower, D. J., Sherratt, E. & Goswami, A. Morphological evolution and modularity of the caecilian skull. *BMC Evol. Biol.* **19**, 1–23 (2019).
2. Marshall, A. F. *et al.* High-density three-dimensional morphometric analyses support conserved static (intraspecific) modularity in caecilian (Amphibia: Gymnophiona) crania. *Biol. J. Linn. Soc.* **126**, 721–742 (2019).
3. Ramírez-Chaves, H. E. *et al.* Mammalian development does not recapitulate suspected key transformations in the evolutionary detachment of the mammalian middle ear. *Proc. R. Soc. B Biol. Sci.* **283**, 20152606 (2016).
4. Sakai, S. T., Whitt, B., Arsznov, B. M. & Lundrigan, B. L. Endocranial Development in the Coyote (Canis latrans) and Gray Wolf (Canis lupus): A Computed Tomographic Study. *Brain. Behav. Evol.* **91**, (2018).
5. Geiger, M. *et al.* Neomorphosis and heterochrony of skull shape in dog domestication. *Sci. Rep.* **7**, 1–9 (2017).
6. Feigin, C. Y. *et al.* Genome of the Tasmanian tiger provides insights into the evolution and demography of an extinct marsupial carnivore. *Nat. Ecol. Evol.* **2**, 182–192 (2018).
7. Spiekman, S. N. F. & Werneburg, I. Patterns in the bony skull development of marsupials: High variation in onset of ossification and conserved regions of bone contact. *Sci. Rep.* **7**, 1–11 (2017).
8. Newton, A. H. *et al.* Letting the ‘cat’ out of the bag: Pouch young development of the extinct tasmanian tiger revealed by X-ray computed tomography. *R. Soc. Open Sci.* **5**, 171914 (2018).
9. Goswami, A. Cranial modularity shifts during mammalian evolution. *Am. Nat.* **168**, 270–280 (2006).
