# Supplementary material for: Ontogenetic origins of cranial convergence between the extinct marsupial thylacine and placental gray wolf
Source: Commun Biol. 2021 Jan 8;4:51. doi: 10.1038/s42003-020-01569-x (PMC7794302; doi:10.1038/s42003-020-01569-x)
Supplement: Supplementary file 3 — Description of Additional Supplementary Files [file 42003_2020_1569_MOESM3_ESM.pdf]

Description of Additional Supplementary Files

File name: Supplementary data 1
Description: List of specimens used in the study. Specimen accession IDs, skull lengths (CBL, mm), dentition patterns and corresponding growth stages are included. Specimens were obtained from published sources (see [3–8]), publically available, and in-house sources.

Supplementary data 2
Description: Cranial landmark locations and module hypotheses from [9]. Landmarks were applied in the forms of points, sliding-landmarks and patches.

File name: Supplementary data 3
Description: Raw ontogenetic landmark coordinates. TPS file format.

File name: Supplementary data 4
Description: Associated taxonomic class, order, family, species, diet and simplified age class classifier variables for raw landmark coordinates.

File name: Supplementary data 5
Description: Associated cranial length (CBL and logCBL) covariates for raw landmark coordinates.
