# Supplementary material for: Ontogenetic origins of cranial convergence between the extinct marsupial thylacine and placental gray wolf
Source: Commun Biol. 2021 Jan 8;4:51. doi: 10.1038/s42003-020-01569-x (PMC7794302; doi:10.1038/s42003-020-01569-x)
Supplement: Supplementary file 9 — Reporting Summary [file 42003_2020_1569_MOESM9_ESM.pdf]

nature research

Corresponding author(s): Christy Hipsley, Axel Newton

Last updated by author(s): Oct 16, 2020

Reporting Summary

Nature Research wishes to improve the reproducibility of the work that we publish. This form provides structure for consistency and transparency in reporting. For further information on Nature Research policies, see our Editorial Policies and the Editorial Policy Checklist.

Statistics

For all statistical analyses, confirm that the following items are present in the figure legend, table legend, main text, or Methods section.

n/a	Confirmed	
☐	☒	The exact sample size (*n*) for each experimental group/condition, given as a discrete number and unit of measurement
☒	☐	A statement on whether measurements were taken from distinct samples or whether the same sample was measured repeatedly
☐	☒	The statistical test(s) used AND whether they are one- or two-sided<br>*Only common tests should be described solely by name; describe more complex techniques in the Methods section.*
☐	☒	A description of all covariates tested
☒	☐	A description of any assumptions or corrections, such as tests of normality and adjustment for multiple comparisons
☐	☒	A full description of the statistical parameters including central tendency (e.g. means) or other basic estimates (e.g. regression coefficient) AND variation (e.g. standard deviation) or associated estimates of uncertainty (e.g. confidence intervals)
☒	☐	For null hypothesis testing, the test statistic (e.g. F, t, r) with confidence intervals, effect sizes, degrees of freedom and P value noted<br>*Give P values as exact values whenever suitable.*
☒	☐	For Bayesian analysis, information on the choice of priors and Markov chain Monte Carlo settings
☒	☐	For hierarchical and complex designs, identification of the appropriate level for tests and full reporting of outcomes
☒	☐	Estimates of effect sizes (e.g. Cohen's d, Pearson's r), indicating how they were calculated

Our web collection on statistics for biologists contains articles on many of the points above.

Software and code

Policy information about availability of computer code

Data collection	Data was collected in the form of 3D landmarks on cranial surface meshes (ply file format) using Landmark Editor (IDAV). Data was exported as raw landmark co-ordinates in NTSYS format for subsequent analyses.
Data analysis	Raw landmark co-ordinates were loaded into MorphoJ from PCA and regression analysis, and Geomorph (R) for allometry and module co-variation analyses.

For manuscripts utilizing custom algorithms or software that are central to the research but not yet described in published literature, software must be made available to editors and reviewers. We strongly encourage code deposition in a community repository (e.g. GitHub). See the Nature Research guidelines for submitting code & software for further information.

Data

Policy information about availability of data

All manuscripts must include a data availability statement. This statement should provide the following information, where applicable:

- Accession codes, unique identifiers, or web links for publicly available datasets
- A list of figures that have associated raw data
- A description of any restrictions on data availability

All CT data generated for this study is publicly available on MorphoSource (www.morphosource.org; project number P1124).

Field-specific reporting

Please select the one below that is the best fit for your research. If you are not sure, read the appropriate sections before making your selection.

☐ Life sciences ☐ Behavioural & social sciences ☒ Ecological, evolutionary & environmental sciences

For a reference copy of the document with all sections, see nature.com/documents/nr-reporting-summary-flat.pdf

Ecological, evolutionary & environmental sciences study design

All studies must disclose on these points even when the disclosure is negative.

Study description	We generated 3D digital cranial models for the thylacine, wolf, dunnart, eastern quoll, koala, brushtail possum and woylie across various stages of development to examine the onset of cranial convergence between the thylacine and wolf.
Research sample	The final data set consisted of 100 individuals spanning the postnatal growth period of each species - thylacine = 22; wolf = 35; dunnart = 11; quoll = 6; koala = 13; possum = 7; woylie = 7
Sampling strategy	We aimed to sample individuals to cover the complete developmental growth trajectory of each species. Species were first sampled after the majority of cranial sutures had visibly closed, but were yet to erupt teeth. These were classified as neonates. On the other end, adults were classified as individuals that showed complete eruption of their adult dentition. We then aimed to sample as many individuals as possible between the neonatal and adult stages. These were classified as either juvenile (eruption of one or many teeth) or subadult (eruption of all but one adult tooth).
Data collection	All the data in this study was collected as 3D digital cranial models. Models were acquired from published studies (see references in main text), public repositories (Digimorph, MorphoSource, Digital Morphology Museum), or were newly generated in-house using X-ray computed tomography (CT).
Timing and spatial scale	The data used in this study was collected over a period from 2017-2019. We first generated adult marsupial cranial models in 2017 for the publication Feigin et al. 2018. We then generated thylacine pouch young CT scans in 2017-2018 for the publication Newton et al. 2018. We next generated additional thylacine models and sourced wolf crania, and sourced marsupial developmental series in 2019 for this study.
Data exclusions	We chose to exclude the youngest thylacine pouch young specimen (DZCU8021) from this study due to the poor quality of the scanned specimen, and incomplete closure of cranial sutures.
Reproducibility	N/A
Randomization	Samples were allocated into developmental stages based largely on their dentition patterns. We chose to specify specimen groups based on the presence of no teeth (neonate), some teeth (juvenile), most teeth (subadult) or all teeth (adult). We chose not to use CBL to group specimens, as this was not a good predictor of developmental stage.
Blinding	*Describe the extent of blinding used during data acquisition and analysis. If blinding was not possible, describe why OR explain why blinding was not relevant to your study.*

Did the study involve field work? ☐ Yes ☒ No

Reporting for specific materials, systems and methods

We require information from authors about some types of materials, experimental systems and methods used in many studies. Here, indicate whether each material, system or method listed is relevant to your study. If you are not sure if a list item applies to your research, read the appropriate section before selecting a response.

Materials & experimental systems

n/a	Involved in the study	
☒	☐	Antibodies
☒	☐	Eukaryotic cell lines
☐	☒	Palaeontology and archaeology
☒	☐	Animals and other organisms
☒	☐	Human research participants
☒	☐	Clinical data
☒	☐	Dual use research of concern

Methods

n/a	Involved in the study	
☒	☐	ChIP-seq
☒	☐	Flow cytometry
☒	☐	MRI-based neuroimaging

Palaeontology and Archaeology

Specimen provenance	Specimens in this study were sourced from Museums Victoria (Australia) and loaned with authorization; and Univeristy of Alaska museum (United States) which were loaned and transported to Australia with authorization from collection managers.
Specimen deposition	All CT data generated for this study is publicly available on MorphoSource (www.morphosource.org; project number P1124).
Dating methods	No new dates are provided.

☐ Tick this box to confirm that the raw and calibrated dates are available in the paper or in Supplementary Information.

Ethics oversight	No special ethical considerations were necessary in this study. Taxon sampling was performed in accordance with relevant guidelines and regulations.

Note that full information on the approval of the study protocol must also be provided in the manuscript.
